# Occurrence and preventability of adverse events in hospitals: a retrospective study

**DOI:** 10.1590/0034-7167-2022-0025

**Published:** 2023-07-10

**Authors:** Antônio José de Lima, Ariane Cristina Barboza Zanetti, Bruna Moreno Dias, Andrea Bernardes, Francielly Marques Gastaldi, Carmen Silvia Gabriel

**Affiliations:** I Universidade de São Paulo. Ribeirão Preto, São Paulo, Brazil; II Hospital de clínicas de Uberlândia. Uberlândia, Minas Gerais, Brazil

**Keywords:** Patient Safety, Medical Errors, Hospitals, Patient Care, Retrospective Studies, Seguridad del Paciente, Errores Médicos, Hospitales, Atención al Paciente, Estudios Retrospectivos, Segurança do Paciente, Erros Médicos, Hospitais, Assistência ao Paciente, Estudos Retrospectivos

## Abstract

**Objectives:**

to analyze the incidence of preventable adverse events related to health care in adult patients admitted to public hospitals in Brazil.

**Methods:**

observational, analytical, retrospective study based on medical records review.

**Results:**

medical records from 370 patients were evaluated, 58 of whom had at least one adverse event. The incidence of adverse events corresponded to 15.7%. Adverse events were predominantly related to healthcare-related infection (47.1%) and procedures (24.5%). Regarding the adverse event severity, 13.7% were considered mild, 51.0% moderate, and 35.3% severe. 99% of adverse events were classified as preventable. Patients admitted to the emergency room had a 3.73 times higher risk for adverse events.

**Conclusions:**

this study’s results indicate a high incidence of avoidable adverse events and highlight the need for interventions in care practice.

## RESUMO

**Objetivos:**

analisar a incidência de eventos adversos evitáveis relacionados ao cuidado em saúde em pacientes adultos internados em hospitais públicos brasileiros.

**Métodos:**

estudo observacional, analítico, de corte retrospectivo, baseado na revisão de prontuários.

**Resultados:**

avaliaram-se prontuários de 370 pacientes, dos quais 58 sofreram pelo menos um evento adverso. A incidência de eventos adversos correspondeu a 15,7%. Os eventos adversos foram vinculados: à infecção relacionada à assistência à saúde (47,1%) e a procedimentos (24,5%), predominantemente. No que tange à gravidade dos eventos adversos, averiguou-se que 13,7% foram considerados leves, 51,0%, moderados e 35,3%, graves. Classificou-se como evitáveis 99% dos eventos adversos. Pacientes internados em caráter de urgência apresentaram risco 3,73 vezes maior para a ocorrência de um evento adverso.

**Conclusões:**

os resultados deste estudo apontam elevada incidência de eventos adversos evitáveis e contribuem para evidenciar a necessidade de intervenções na prática assistencial.

## RESUMEN

**Objetivos:**

analizar la incidencia de eventos adversos evitables relacionados al cuidado de la salud en pacientes adultos internados en hospitales públicos brasileños.

**Métodos:**

estudio observacional, analítico, retrospectivo, basado en la revisión de historias clínicas.

**Resultados:**

se evaluaron las historias clínicas de 370 pacientes, 58 de los cuales sufrieron al menos un evento adverso. La incidencia de eventos adversos correspondió al 15,7%. Los eventos adversos estaban relacionados, principalmente, con: infecciones por asistencia sanitaria (47,1%) y procedimientos (24,5%). Respecto a la gravedad de los eventos adversos, el 13,7% era leve, el 51%, moderado y el 35,3%, grave. Se clasificó como evitable el 99% de los eventos adversos. Los pacientes ingresados en urgencias presentaron un riesgo 3,73 veces mayor de aparición de eventos adversos.

**Conclusiones:**

los resultados de este estudio señalan una incidencia elevada de eventos adversos evitables y resaltan la necesidad de intervenciones en la práctica asistencial.

## INTRODUCTION

The World Health Organization (WHO) Global Patient Safety Action Plan 2021-2030 points out that failures in patient safety are recognized as a major and growing global public health challenge and are one of the leading causes of death and disability worldwide^(1)^. Patient safety actions had a variable impact and much of it has not been adjusted for successful implementation in low and middle-income countries^(2)^.

On average, an estimated one in 10 patients is subject to an adverse event while receiving hospital care in high-income countries^(1)^. The estimate for low and middle-income countries suggests that as many as one in four patients is harmed, with 134 million adverse events occurring annually due to unsafe care in hospitals, contributing to an estimated 2.6 million deaths^(3)^. Most patient harm is preventable^(1)^.

The World Health Organization (WHO) defines patient safety as a framework of organized activities that creates health care culture, processes and procedures, behaviors, technologies, and environments which consistently and sustainably reduce risk, avoidable harm, error probability, and its impact when it occurs^(1)^.

Adverse Event (AE) is defined as an unintentional injury or damage resulting in disability or dysfunction, temporary or permanent, and/or prolongation of hospital stay or death as a result of the health care provided, with no link to the underlying disease process of the patient. When classified as preventable, the adverse event is defined as harm to the patient associated with an active failure or a latent condition, or even a violation of norms and standards^(4-5)^. Approximately 12% of preventable AEs cause permanent disability or patient death and are mainly related to incidents with medications, therapeutic management, and invasive procedures^(6)^.

Identifying AEs that are considered preventable can be one of the ways to improve patient safety, and is one of the strategies proposed by the WHO, which highlights the need for analysis of AE data to generate learning in health systems and services^(1)^.

To identify AEs in hospitals, retrospective chart review has been used since The Harvard Medical Practice Study (HMPS), a forerunner of other tools such as the Assessment Tool developed by the Quality in Australian Health Care Study (QAHCS) and the Assessment Tool developed by the Canadian Adverse Event Study (CAES)^(7)^.

In Brazil, the National Program for Patient Safety^(8)^ points out the need for epidemiological studies that portray the extent of the AE problem in different Brazilian regions, taking into account the heterogeneity of our healthcare institutions.

## OBJECTIVES

To analyze the incidence of preventable adverse events related to health care in adult patients admitted to Brazilian public hospitals.

## METHODS

### Ethical Aspects

The study was approved by the Research Ethics Committees of the proposing institution and the participating institution. To ensure its security, the data were deposited in a single database on the server allocated at the research proponent institution. All medical record evaluators were trained and signed a term related to the need to maintain data confidentiality.

### Study design, period and setting

This is an observational, analytical, retrospective study, with a medical record review of patients admitted to two general, public, and teaching hospitals that provide low, medium, and high complexity services, identified as hospital A with 506 beds, and hospital B with 235 beds. Data collection was conducted in the period between March 2019 and February 2020. This study follows the Strengthening the Reporting of Observational Studies in Epidemiology (STROBE) recommendations.

### Population, sample, and inclusion and exclusion criteria

The study population comprised a total of 14,753 hospitalizations. For the sample calculation, we considered a probability of 8.6% for AE occurrence, a 95% confidence level and a 3% absolute error, and an estimated loss of 10%; considering stratified sampling proportional to the admissions in each of the two hospitals. The sample calculation resulted in 370 medical records, 202 in hospital A and 168 in hospital B.

We considered eligible all adult patient admissions that occurred in 2015, except for admissions for obstetric, psychiatric, and palliative care causes.

### Study protocol

The study was conducted in two phases, screening and evaluation, using a computerized version of the protocol developed by the researchers responsible for the Iberoamerican Study of Adverse Events (IBEAS)^(9-10)^, which was pioneered by The Harvard Medical Practice Study (HMPS)^(11)^ and the Canadian Adverse Event Study (CAES)^(12)^, adapted and validated for the reality of Brazilian hospitals^(13)^.

In the screening phase, performed by two nurses, the presence of potential adverse events (pAE) was identified, as well as the demographic, clinical, and inpatient profile delineation. Identifying adverse events occurs from the selection of at least one of the 19 screening criteria^(9,13)^ (TABLE 1). The existence of pAE in this phase has selected the medical records for the second phase of evaluation, carried out by two physicians, which consisted in evaluating the medical records to identify AEs, background, characterization, causal factors, contributors, and the possibility of avoidability.

**Chart 1:** Screening criteria for potential adverse events

SCREENING CRITERIA
1. Prior hospitalization in the past 12 months in patients younger than 65, or prior hospitalization in the past 6 months in patients 65 and older.
2. Antineoplastic treatment in the 6 months prior to hospitalization.
3. Trauma, accident, or fall during hospitalization.
4. Unwanted medication effect.
5. Fever above 38.3º C on the day before scheduled discharge.
6. Transfer from a general inpatient unit to an intensive or semi-intensive care unit.
7. Transfer to another acute care hospital.
8. Second surgical intervention during this hospitalization.
9. After performing an invasive procedure, an injury has occurred to an organ or system that will require clinical or surgical treatment.
10. Neurological change missing on admission but present during the study period.
11. AMI (acute myocardial infarction), stroke, or PTE (pulmonary thromboembolism) during or after an invasive procedure.
12. Cardiac arrest or low APGAR score
13. Injury or complication related to abortion, amniocentesis, labor, or prepartum.
14. Death
15. Unplanned open surgical intervention or admission for intervention, whether laparoscopic or open, after a scheduled outpatient intervention.
16. Some injury or complication related to an outpatient surgery or invasive procedure that resulted in hospitalization or evaluation in the emergency department.
17. Any type of infection associated with care.
18. Documentation or correspondence in the medical record (including a property claim) regarding the care that could suggest litigation.
19. Any other unwanted occurrence not mentioned above.

**Table 1:** Association between categorical variables (demographic, clinical, and hospitalization) and adverse events occurrence in two general, public, and teaching hospitals (n=80), Uberlândia, Minas Gerais, Brazil, 2021

Variables	Adverse event				Total (n=80)		*p* value
	Present (n=58)		Absent (n=22)				
	n	%	n	%	n	%	
Gender							0.897^*^
Male	37	63.8	13	59.1	50	62.5	
Female	21	36.2	9	40.9	30	37.5	
Age group (years)							1.000^*^
<60	31	53.4	12	54.5	43	53.8	
≥60	27	46.6	10	45.5	37	46.2	
Education							0.781^**^
Incomplete elementary school	6	10.3	0	0.0	6	7.5	
Complete elementary school	23	39.7	4	18.2	27	33.7	
Complete High School	3	5.2	0	0.0	3	3.8	
Incomplete high school	1	1.7	0	0.0	1	1.2	
Complete higher education	3	5.2	0	0.0	3	3.8	
None	10	17.2	0	0.0	10	12.5	
No information	12	20.7	18	81.8	30	37.5	
Race							0.448^**^
White	23	39.7	10	45.5	33	41.2	
Black	4	6.9	3	13.6	7	8.8	
Brown (Pardo)	31	53.4	9	40.9	40	50.0	
Comorbidity							1.000^**^
Absent	8	13.8	3	13.6	11	13.8	
Present	50	86.2	19	86.4	69	86.2	
CID-10 Chapters							0.015^**^
I	12	20.7	0	0.0	12	15.0	
II	3	5.2	4	18.2	7	8.8	
VI	1	1.7	1	4.5	2	2.6	
IX	11	19.0	0	0.0	11	13.7	
X	3	5.2	2	9.1	5	6.2	
XI	5	8.6	3	13.6	8	10.0	
XII	1	1.7	0	0.0	1	1.2	
XIII	0	0.0	1	4.5	1	1.2	
XIV	5	8.6	3	13.6	8	10.0	
XVIII	1	1.7	1	4.5	2	2.6	
XIX	15	25.9	7	31.9	22	27.5	
XXI	1	1.7	0	0.0	1	1.2	
Intrinsic risk factors							0.541^**^
Absente	12	20.7	3	13.6	15	18.7	
Present	46	79.3	19	86.4	65	81.3	
Extrinsic risk factors							-
Absente	0	0.0	0	0.0	0	0.0	
Present	58	100	22	100	80	100	
Prognosis of the disease motivating hospitalization:							
Full recovery to baseline health							0.033^**^
No	35	60.3	19	86.4	54	67.5	
Yes	23	39.7	3	13.6	26	32.5	
Recovery with residual disability							0.772^**^
No	15	25.9	5	22.7	20	25.0	
Yes	43	74.1	17	77.3	60	75.0	
Terminal illness							0.283^*^
No	52	89.7	17	77.3	69	86.3	
Yes	6	10.3	5	22.7	11	13.7	
Hospitalization type							0.033^*^
Urgency	49	84.5	13	59.1	62	77.5	
Elective	9	15.5	9	40.9	18	22.5	
Hospital exit type							0.889^**^
Discharge	41	70.7	15	68.2	55	68.7	
Death ≤24h	2	3.4	0	0.0	2	2.5	
Death >24h	15	25.9	7	31.8	23	28.8	
Hospitalization time (days)							0.615^**^
<3	11	19.0	2	9.1	13	16.3	
3 to 10	15	25.9	6	27.3	21	26.2	
>10	32	55.1	14	63.6	46	57.5	
Hospitalization sector							0.038^**^
Clinical	10	17.2	4	18.2	14	17.5	
Surgical	27	46.6	16	72.7	43	53.8	
Intensive Care	21	36.2	2	9.1	23	28.7	

* Pearson's chi-square test;

** Fisher's exact test.

The AE identification phase is based on a six-level identification scale, ranging from (1) absence, (2) minimal, (3) low, (4) moderate, (5) very likely to (6) total evidence that the injury or damage was a consequence of the health care provided^(9,13)^. And the avoidability of the AE is evaluated according to a six-degree evidence scale that the injury or damage could be avoided, in which (1) no evidence, (2) minimal, (3) mild, (4) moderate, (5) high evidence to (6) total evidence that the injury or damage could be avoided^(9,13)^. In both scales, responses from four to six correspond to confirmation of the presence of AE and avoidability of AE, respectively.

### Results analysis and statistics

In this retrospective study, the incidence of patients with AE was obtained by the ratio between the patients presenting at least one AE over the overall number of patients in the study; the AE incidence density, in the analyzed period, was obtained by the ratio between the numbers of patients presenting at least one AE and the cumulative number of hospital stay days of all patients in the study multiplied by 100; and the avoidable AE proportion was obtained by the ratio between the number of avoidable AEs over the total number of AEs.

For the statistical analyses, processed using IBM SPSS Statistics, version 26.0, and R Core Team (2020) software, we considered the significance level α=0.05 and 95% confidence interval (CI).

Descriptive statistics were used for all study variables to characterize the sample. Pearson’s Chi-square and Fisher’s exact tests were used to verify the associations between the explanatory variables: gender; age group; education; race; presence of comorbidity; ICD-10 chapters; intrinsic and extrinsic risk factors; prognosis of the disease that motivated hospitalization; hospitalization type; discharge type; hospitalization time; and hospitalization sector - with the dependent variable AE presence; as well as to verify the association between the explanatory variables: AE classification; AE avoidability; presence of comorbidity; inpatient sector; and disease prognosis that motivated the admission - with the dependent variable AE severity. The selection of these variables was in accordance with the findings of previous AE studies^(10,14)^ and the protocol developed by the researchers responsible for the Iberoamerican Adverse Events Study (IBEAS)^(9-10)^ used as a methodological reference for this research.

The standard logistic regression model was adopted (by GLM class) aiming to test the strength of different variables on a given outcome, in this case, the presence or not of AE. As independent variables for the AE presence were considered the reference variables: Hospital (A), Gender (Male), Age (< 60), Education (High School or College), Comorbidity (Absent), Hospitalization Type (Elective), Inpatient Sector (Clinical) and ICD-10 Chapters (II, VI, XII, XIII, XVIII, XXI).

The selection of independent variables was carried out by the Likelihood Ratio test. Due to the small number of independent variables evaluated, it was not necessary to apply the automatic selection procedures *(backward, forward)*. For the significant parameters of the adjusted model, we calculated the respective odds ratios (Odds Ratio).

## RESULTS

**Figure 1:**
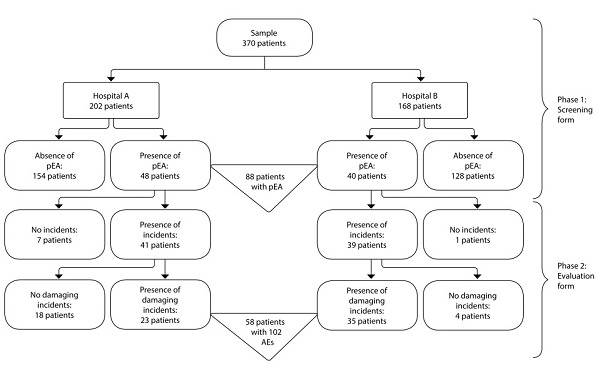
Flow of retrospective medical record review

We analyzed the medical records of 370 patients admitted to the two hospitals, among whom 88 had potential adverse events (pAE), making them eligible for the second phase of the study. Phases of the retrospective chart review are detailed in Figure 1.

In this study, we observed that 58 (15.7%) patients experienced 102 healthcare-related AEs, an AE incidence density of 1.89 per 100 patient-days. Table 1 presents the demographic, clinical, and hospitalization characteristics of patients with incident records.

The variables: ICD-10 Chapters (p=0.015), Prognosis - complete recovery (p=0.033), Hospitalization Type (p=0.033), and Hospital Sector (p=0.038) were associated with the AE occurrence.

AEs, according to the main problem investigated, were classified as: related to healthcare-associated infection (HAI) (47.1%); procedure-related (24.5%); care-related (14.7%); medication-related (5.9%); diagnosis-related (3.9%); and other AE types (3.9%).

Characterization as preventable AE was identified in 99% of AEs. Death occurred in 29.3% of patients who suffered some AE, out of which 94.2% had evidence of association or causality between the AE and death.

The variable Prognosis of the disease leading to hospitalization - Full recovery (p<0.001) showed an association with AE severity (Table 2).

**Table 2:** Associations between the variables classification, avoidability, comorbidity, sector, and prognosis of the disease that motivated the admission and the severity of adverse events in patients from two general, public, and teaching hospitals (n=102), Uberlândia, Minas Gerais, Brazil, 2021

Variables	Adverse event Severity						Total		*p* value
	Mild		Moderate		Severe				
	n	%	n	%	N	%	N	%	
AE Classification									0,545^**^
Related to general care	5	33.3	5	33.3	5	33.3	15	100	
Related to medication	0	0.0	4	66.7	2	33.3	6	100	
Related to hospital infection	7	14.6	25	52.1	16	33.3	48	100	
Related to the procedure	2	8.0	14	56.0	9	36.0	25	100	
Related to diagnosis	0	0.0	1	25.0	3	75.0	4	100	
Others	0	0.0	3	75.0	1	25.0	4	100	
Avoidability of AE									1.000^**^
Not avoidable	0	0.0	1	100	0	0.0	1	100	
Avoidable	14	13.9	51	50.5	36	35.6	101	100	
Comorbidity									0.105^**^
Absent	3	37.5	2	25.0	3	37.5	8	100	
Present	11	11.8	49	52.7	33	35.5	93	100	
Hospitalization sector									0.079^**^
Clinical	3	12.5	15	62.5	6	25.0	24	100	
Surgical	8	22.2	12	33.3	16	44.5	36	100	
Intensive/Semi-intensive Care	3	7.2	25	59.5	14	33.3	42	100	
Prognosis of the disease motivating the hospitalization:									
Full recovery to baseline health									0.000^**^
No	3	4.2	38	53.5	30	42.3	71	100	
Yes	11	35.5	14	45.2	6	19.3	31	100	
Recovery with residual disability									0.341^*^
No	7	12.1	27	46.5	24	41.4	58	100	
Yes	7	15.9	25	56.8	12	27.3	44	100	
Terminal illness									0.595^**^
No	12	13.5	47	52.8	30	33.7	89	100	
Yes	2	15.4	5	38.5	6	46.1	13	100	

* Pearson's chi-square test;

** Fisher's exact test.

Regarding the AE consequences, we found that AEs caused an increase in the length of hospital stay by 65.7% and, furthermore, 11.8% of the AEs were responsible for readmission. In this sense, 846 days of hospitalization were added, with a mean of 10.7 days per event.

AE incidence was different among hospitals, in which hospital and type of admission were identified as risk factors for AE occurrence (Table 3).

**Table 3:** Logistic regression analysis of factors regarding demographic, clinical, and hospitalization characteristics associated with the risk of adverse events in patients from two public general and teaching hospitals (n=80), Uberlândia, Minas Gerais, Brazil, 2021

Variables	Adverse event		Crude OR (95% CI)	Crude *p* value	Adjusted OR (95% CI)	Adjusted *p* value
	Present n (%)	Absent n (%)				
Hospital						
A	23 (39.7)	18 (81.8)	Ref	Ref	Ref	Ref
B	35 (60.3)	4 (18.2)	6.85 (2.05.22.83)	0.002	6.8 (1.97.23.55)	0.002
Gender						
Male	37 (63.8)	13 (59.1)	Ref	Ref	-	-
Female	21 (36.2)	9 (40.9)	0.82 (0.3.2.24)	0.698	-	-
Age group (years)						
<60	31 (53.4)	12 (54.5)	Ref	Ref	-	-
≥60	27 (46.6)	10 (45.5)	1.05 (0.39.2.8)	0.930	-	-
Education					-	-
Elementary school complete or incomplete	29 (50.0)	4 (18.2)	0 (0.Inf)	0.995	-	-
High school complete or incomplete or higher education	7 (12.1)	0 (0.0)	Ref	Ref	-	-
None	10 (17.2)	0 (0.0)	1 (0.Inf)	1.000	-	-
No information	12 (20.7)	18 (81.8)	0 (0.Inf)	0.994	-	-
Comorbidity						
Absent	8 (13.8)	3 (13.6)	Ref	Ref	-	-
Present	50 (86.2)	19 (86.4)	0.99 (0.24.4.12)	0.985	-	-
ICD-10 Chapters						
I	12 (20.7)	0 (0.0)	115648793.27 (0.Inf)	0.992	-	-
IX. X. XI. XIV	24 (41.4)	8 (36.3)	1.91 (0.53.6.84)	0.321	-	-
XIX	15 (25.9)	7 (31.9)	4.5 (1.20.31)	0.050	-	-
II. VI. XII. XIII. XVIII. XXI	7 (12.0)	7 (31.8)	Ref	Ref	-	-
Hospitalization type						
Urgency	49 (84.5)	13 (59.1)	3.77 (1.24.11.41)	0.019	3.73 (1.1.12.61)	0.034
Elective	9 (15.5)	9 (40.9)	Ref	Ref	Ref	Ref
Hospitalization sector						
Clinical	10 (17.2)	4 (18.2)	Ref	Ref	-	-
Surgical	27 (46.6)	16 (72.7)	0.67 (0.18.2.51)	0.558	-	-
Intensive/Semi-intensive Care	21 (36.2)	2 (9.1)	4.2 (0.66.26.9)	0.130	-	-

*OR - Odds Ratio; ref - reference category. ICD-10 Chapters: I - Some infectious and parasitic diseases; II - Neoplasms [tumors]; VI - Nervous system diseases; IX - Circulatory system diseases; X - Respiratory system diseases; XI - Digestive system diseases; XII - Skin and subcutaneous tissue diseases; XIII - Musculoskeletal and connective tissue diseases; XIV - Genitourinary system diseases; XVIII - Symptoms, signs and abnormal findings of clinical and laboratory tests, not classified elsewhere; XIX - Injuries, poisoning, and some other external causes consequences; XXI - Factors that influence health status and contact with health services.*

## DISCUSSION

Identifying the incidence and characteristics of AEs in health services is essential to recognize the scale of the problem, as well as propose actions to mitigate new AEs. The AE incidence rate found in this study was 15.7% and an AE incidence density of 1.89 per 100 patient-days. Studies conducted in Brazil with similar scope present different rates, ranging from an AE incidence rate of 8.6%, with an AE incidence density of 0.8 per 100 patient-days^(15)^ to an AE incidence rate of 33.7%, and AE incidence density of 4.97 per 100 patient-days^(7)^.

The incidence rate may vary depending on the instrument used for the survey, reviewers’ expertise, a sample of the patient population studied, the study setting, the hospital’s organizational safety culture, AE concept and cause range, and the quality of literature reviewed^(16-17)^.

In Brazil, notification of AEs in the national system is mandatory, but far from representing reality. In this sense, it is imperative to advance the involvement and engagement of the population, professionals, and institutions to increase notifications and implement several strategies, such as the methodology adopted in this research, to identify the real size of the AE problem in health services.

The demographic variables (gender, age, education, and race) showed no association with AE occurrence, corroborating the results of another study conducted in Brazil^(7)^, and diverging from a study conducted in Portugal, which highlights age over 60 years as a risk factor for AE occurrence^(18)^.

The elevated percentage of patients with AE and intrinsic risk factors found in this study can demand the need for additional treatments, and increase care complexity since the presence and number of intrinsic and extrinsic risk factors increase the patient’s chance of suffering an AE^(19)^. A Chilean study indicates that among the patients who suffered AE, 58.1% presented intrinsic risk factors, the most recurrent: being arterial hypertension, diabetes, hypoalbuminemia, and obesity^(20)^. In the present study, arterial hypertension was the most commonly found intrinsic risk factor, which may reflect the high prevalence of arterial hypertension in Brazilian adults, especially among those over 60 years of age^(21)^.

Extrinsic risk factors correspond to interventions conducted by health professionals due to the needs of care, such as the use of venous or arterial catheters, urinary catheterization, and in this study, all patients who presented AE had extrinsic risk factors, and these reached up to 12 factors, which denotes high care complexity and the risk of multiple damages, a situation also observed in other studies^(7,20)^.

Patients’ medical diagnoses recorded at admission, the need for interventions, as well as longer hospital stays, may be associated with the occurrence of AEs^(7,20)^. In this study, the main medical diagnosis, identified at admission, according to ICD-10, showed an association with AE occurrence, and this association has not been identified or discussed in other studies^(10,15)^. The medical diagnosis recorded at admission is associated with AE because, in theory, it demands more procedures and technical acts that expose patients to risk, even more so if we consider that moderate and severe AE occurs mainly in the intensive/semi-intensive care and surgical units, with a higher proportion originating from admissions due to external causes, infections, and circulatory system diseases, also respectively.

The prognosis “full recovery” was associated with AE occurrence, but this association was not pointed out or discussed in other studies^(7,9,15)^. It was noted that both in the group of patients with AE and in the group of those without AE, “not full recovery” and “recovery with some residual disability” were the predominant prognoses. On the other hand, patients from a private Chilean hospital^(20)^ presented full recovery as the predominant inpatient prognosis^(22)^.

Regarding AE-related factors, although there is evidence that HAIs are preventable AEs and strategies for their prevention are effective^(23)^, the percentage of AEs related to HAIs corresponds to almost half of the AE-related factors, among which pneumonia occurred in a quarter. Other studies have reported HAI-related AEs ranging from 10.8% to 32.6%^(7,18,20)^.

This AE percentage related to HAIs may be associated with another finding of this study, which was the high avoidability rate of AEs, reaching 99% of AEs, a finding that differs absolutely from other studies, national and international^(7,19-20,24)^.

An AE occurrence and its severity can predict the intensity of the AE repercussion during hospitalization since it may require additional diagnostic tests and/or treatments, increase the length of hospitalization, and generate disability and/or readmission and death. AEs increase the patient’s hospital stay and can cause readmission, as identified in other national and international studies^(7,19,24)^.

In this study, most AEs were classified as moderate or severe, with a consequent increase in length of stay, disability at discharge, or need for surgical intervention. This predominance of AEs classified as moderate and severe may be associated with the researched institutions’ characteristics, as well as the complexity of the sample patients, demonstrated by the presence of comorbidities and intrinsic and extrinsic risk factors.

In this study, death was identified in one-third of patients, a fact that was also observed in a Portuguese study^(19)^. Although the association of death with AE is difficult to be inferred since there are implicit variables in the health care context, related to the patient himself, the health service, and the health teams involved^(23)^, approximately 12% of preventable AEs cause permanent disability or patient death ^(6)^.

Understanding the incidence, nature, severity, avoidability, and risk factors associated with AE occurrence in the hospital environment provides opportunities to implement AE mitigation strategies, as well as to raise a patient safety culture^(2,7)^.

The results of this study ratify different incidence rates among hospitals, as well as the Korean study that identified a more than 10-fold difference in AE incidence and avoidability among hospitals and among departments in the same hospital^(16)^.

It is also suggested that there is a 6.8 times greater risk of an AE occurring in hospital B than in hospital A. However, we should emphasize that it is necessary to evaluate the patient safety conditions and characteristics of each institution^(16)^, considering that AE identification in medical records may be associated with the quality of records and greater patient safety culture.

Safety culture of an organization is the product of values, attitudes, perceptions, skills, and individual and group behavior patterns, which determines the organization’s health and safety management characteristics^(25)^.

The working environment with poor working conditions and the excessive workload of nursing workers may contribute to unsafe care^(26)^.

The conditions of patient safety involve, in addition to protocols and technology, the creation of a psychologically safe work environment, where healthcare workers can talk about patient safety and other concerns without fear or negative consequences, depending on leadership commitment, transparency, open and respectful communication, learning from mistakes and best practices, and a careful balance between a no-blame policy and accountability^(1)^.

Considering the admission type, patients admitted to emergency services had approximately four times higher risk for AEs than those admitted to elective services. However, other studies on AE incidence did not identify any association between the admission type of patients in health services and AE occurrence^(7,14)^.

Patient admission via the emergency department is a factor that adds complexity to care, increasing AE risk, both in terms of patient characteristics and structural and organizational aspects related to the emergency unit’s context. The variability of scenarios in safety studies must be considered since different strategies are required in highly unpredictable scenarios such as emergencies^(27)^.

These findings directly affect the decisions regarding quality improvement in health care for hospital institutions, since they offer data to health professionals, managers, researchers, and educators for the most frequent AE types, their related factors, and avoidability, besides providing subsidies for new policies to face organizational problems, influencing educational programs implementation, contributing to reduce the length of patient’s hospital stay and, consequently, minimizing the financial impact of AEs on the institutions and health system costs.

Reducing AE rates is determined by a combination of factors, namely: health professionals’ attitudes, leaders and managers of health care organizations, policymakers, and the evidence that must be used on an ongoing basis to develop interventions that are incorporated into practice^(23-24,28)^.

### Study Limitations

As study’s limitations, we include those related to the methodological design itself, the evaluator’s subjectivity and clinical impression, the information quality contained in the medical records, and the limitation of the data collection instrument validated in other contexts.

### Contributions to the Area

Identifying AEs in health services is essential to recognize the scenario and support assertive interventions to reduce the risks to patients.

The active search for AEs, retrospective or real-time, aims to fill the gaps in voluntary reporting systems.

Based on this study’s analysis of the results regarding avoidable AE incidents, we highlight how important it is to systematically generate, analyze, and use data on AEs, because despite the development of studies on patient safety and quality in health care context, there is still a weakness in medical records, and in the collection, dissemination, and adoption of data to support efforts to implement changes in care practices and, in particular, in nursing work.

## CONCLUSIONS

Healthcare-related AE incidence in the hospitals studied was 15.7% with a density of 1.89 per 100 patient-days, and 99% could be prevented. AEs were mainly related to healthcare-related infections. Regarding their severity, 86.3% of AEs were considered moderate and severe. We also verified AE occurrence association with ICD-10 chapters: external causes, especially infections and circulatory system diseases; full recovery prognosis; hospitalization type and hospitalization sector. The incidence rates are different among hospitals and that hospital admission by the emergency room presented a higher risk for AE occurrence.

This study’s major findings point to important issues regarding the epidemiology of patient safety, which shows a high incidence and high avoidability of AEs, with severity rates ranging from moderate to high.

Understanding and reducing the numbers of harmful AEs, their causes, and their consequences should be an aim of the leadership and interdisciplinary teams in healthcare services, as it will allow developing organizational models, capable of providing ways to ensure patient and staff safety.
